# Correction: Kim *et al*. Histone Deacetylase Inhibitor, Mocetinostat, Regulates cardiac remodelling and renin-angiotensin system activity in rats with transverse aortic constriction-induced pressure overload cardiac hypertrophy. Reviews in Cardiovascular Medicine. 2021; 22(3): 1037–1045

**DOI:** 10.31083/RCM48594

**Published:** 2025-11-27

**Authors:** Gun Jik Kim, Hanna Jung, Eunjo Lee, Sung Woon Chung

**Affiliations:** ^1^Department of Thoracic and Cardiovascular Surgery, Kyungpook National University Hospital, School of Medicine, Kyungpook National University, 41944 Daegu, Republic of Korea; ^2^Emerging Infectious Disease Vaccines Division, National Institute of Food and Drug Safety Evaluation, Ministry of Food and Drug Safety, 28159 Cheongju, Republic of Korea; ^3^Department of Thoracic and Cardiovascular Surgery, Pusan National University Hospital, Pusan National University School of Medicine, 49241 Busan, Republic of Korea

The author would like to correct Fig. 5 of Histone deacetylase inhibitor, mocetinostat, regulates cardiac remodelling and renin-angiotensin system activity in rats with transverse aortic constriction-induced pressure overload cardiac hypertrophy [1], as errors were introduced in Fig. 5. The author declares that these corrections do not change the result or conclusion of this paper. We sincerely apologize for having this error in the article, and apologize for any inconvenience caused. The authors have provided a corrected version of Fig. 5 here:

**Figure:**
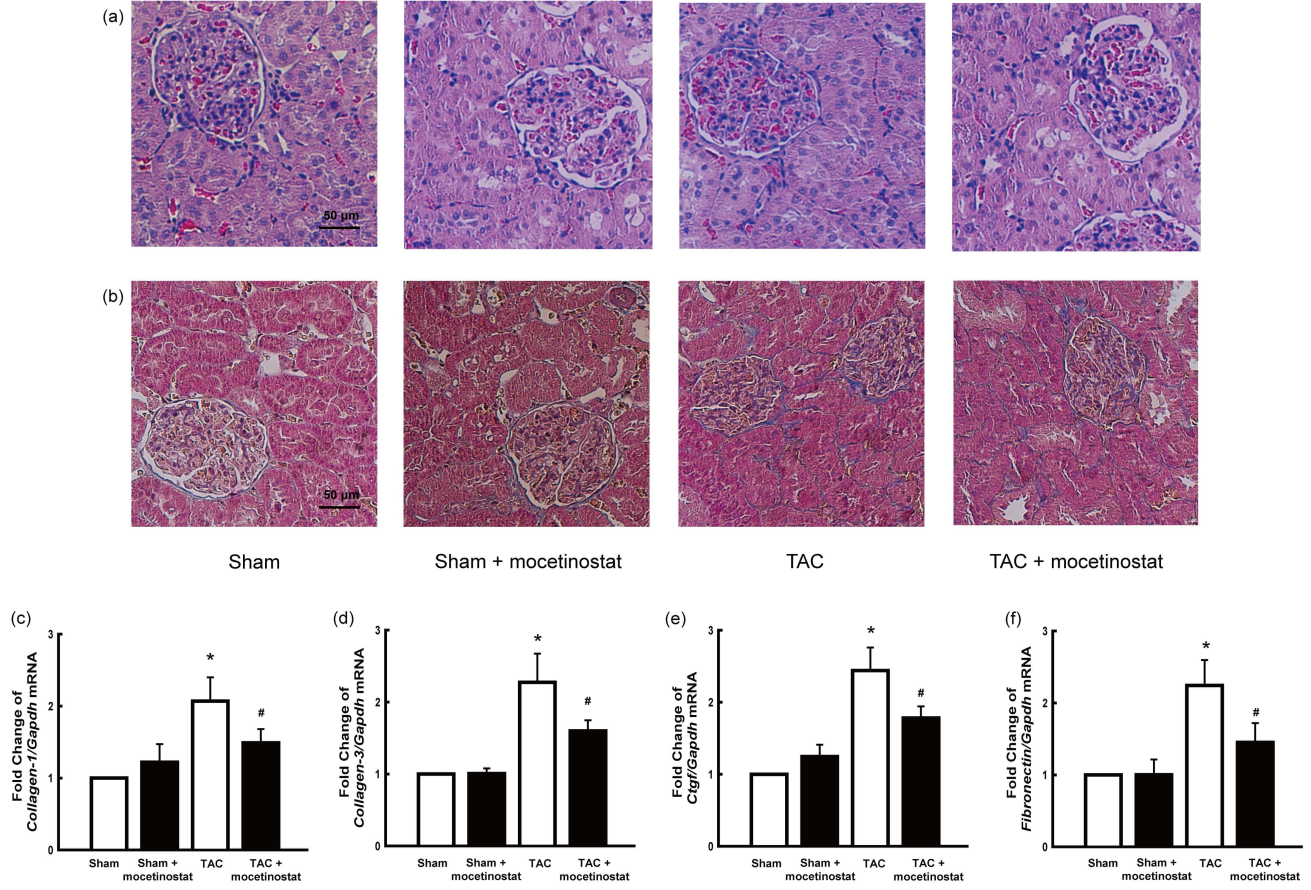
The original Fig. 5.

**Figure:**
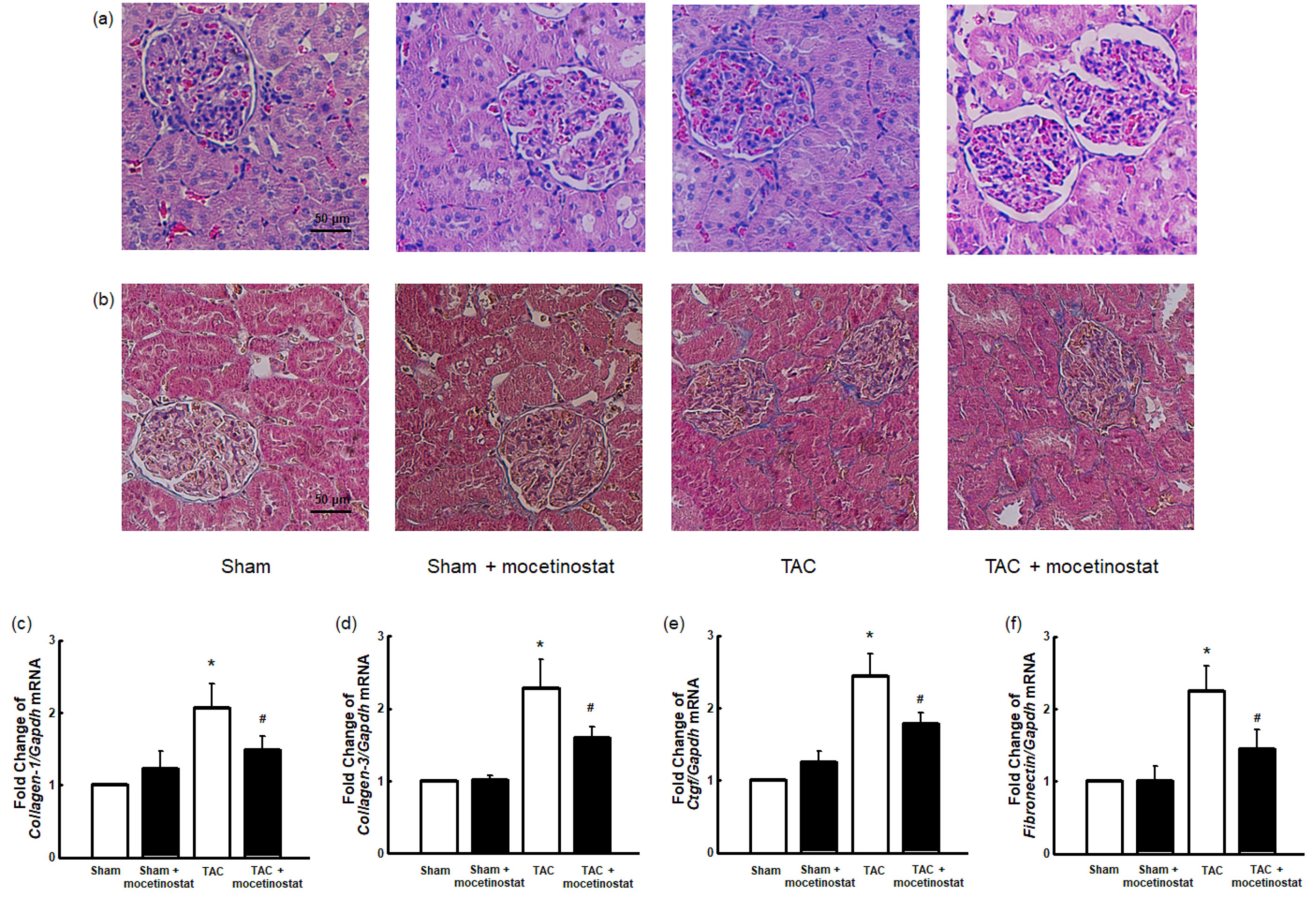
The corrected Fig. 5.

This has been corrected as of 28 October 2025. The authors apologize for these errors. This correction has been approved by the Editor-in-Chief of the journal.

## References

[1] Kim GJ, Jung H, Lee E, Chung SW (2021). Histone deacetylase inhibitor, mocetinostat, regulates cardiac remodelling and renin-angiotensin system activity in rats with transverse aortic constriction-induced pressure overload cardiac hypertrophy. *Reviews in Cardiovascular Medicine*.

